# Bone in the Colon: A Case of Osseous Metaplasia in a Juvenile Colonic Polyp

**DOI:** 10.7759/cureus.112742

**Published:** 2026-07-15

**Authors:** Mira Almheiri, Ehsan Malik, Anas Bernieh

**Affiliations:** 1 Pediatric Gastroenterology, Al Jalila Children's Speciality Hospital, Dubai, ARE; 2 Pediatric Gastroenterology, Al Jalila Children's Specialty Hospital, Dubai, ARE; 3 Anatomic Pathology, Dubai Health, Dubai, ARE

**Keywords:** biopsy, colonoscopy, colon polypectomy, juvenile polyp, osseous metaplasia

## Abstract

Osseous metaplasia is defined as the formation of bone at extra-skeletal sites. The occurrence of osseous metaplasia is rare in the gastrointestinal tract and even less common in the colon; a few reported cases in both adults and pediatrics have been reported. An osseous metaplasia is associated with both benign and malignant diseases, but the exact pathogenesis for this condition is unknown.

A 14-year-old male presented with abdominal pain, alternating bowel habits, and urgency for the past three months, and he lost 5 kg unintentionally. He denies fever, blood in stool, extraintestinal manifestations, and nocturnal symptoms. His past medical and family history is unremarkable. His physical examination was normal, and he had a negative celiac screening, inflammatory markers, and fecal calprotectin. There was minimal improvement with peppermint oil, antispasmodics, and dietary modifications. Colonoscopy: a pedunculated polyp < 5 mm in the transverse colon; polypectomy done with epinephrine injection to elevate the stalk (1:100,000) and hot snare without complication. Histology revealed a juvenile polyp with osseous metaplasia.

We report a rare incidental histological finding in a juvenile polyp that has been reported rarely worldwide with different gastrointestinal lesions. Although the underlying mechanism is unclear, further follow-up is needed to determine the prognosis and its significance.

## Introduction

Osseous metaplasia refers to the formation of mature bone tissue in ectopic locations outside the normal skeletal system and has been described in several organs, including the skin, breast, lung, thyroid, and genitourinary tract [1,2,3]. Within the gastrointestinal tract, osseous metaplasia is particularly rare and has been reported in association with both benign and malignant lesions, including juvenile polyps and colorectal adenocarcinoma [4,5]. However, its exact prevalence and pathogenesis remain poorly understood because most available data are limited to isolated case reports and small case series [1,3,4].

Most reported pediatric cases involved rectal polyps presenting with rectal bleeding [3,4]. We report a rare case of osseous metaplasia identified within a juvenile polyp located in the transverse colon of a 14-year-old boy presenting primarily with abdominal pain and altered bowel habits. This case highlights an unusual histopathological finding in an uncommon anatomical location.

## Case presentation

A 14-year-old Emirati boy with a three-month history of progressive abdominal pain. The pain was generalized, occurred almost daily, and was relieved by defecation. His symptoms affected his daily activities and resulted in missed school days. The pain was associated with urgency and diarrhea; however, he denied nocturnal symptoms, hematochezia, or extraintestinal manifestations, such as fever, rash, or joint pain. He lost around 5 kg during the preceding month.

His antenatal, past medical, and family histories were unremarkable. Vital signs were within normal limits. His vitals were within the normal range for age, and the physical examination showed no clubbing, pallor, oral ulcers, abdominal tenderness, or perianal abnormalities.

Laboratory investigations, including complete blood count, C-reactive protein, erythrocyte sedimentation rate, anti-tissue transglutaminase antibodies, thyroid function tests, fecal calprotectin, and abdominal ultrasound, were all unremarkable. A trial of peppermint oil and antispasmodic therapy for presumed irritable bowel syndrome was initiated; however, his symptoms persisted.

**Figure 1:**
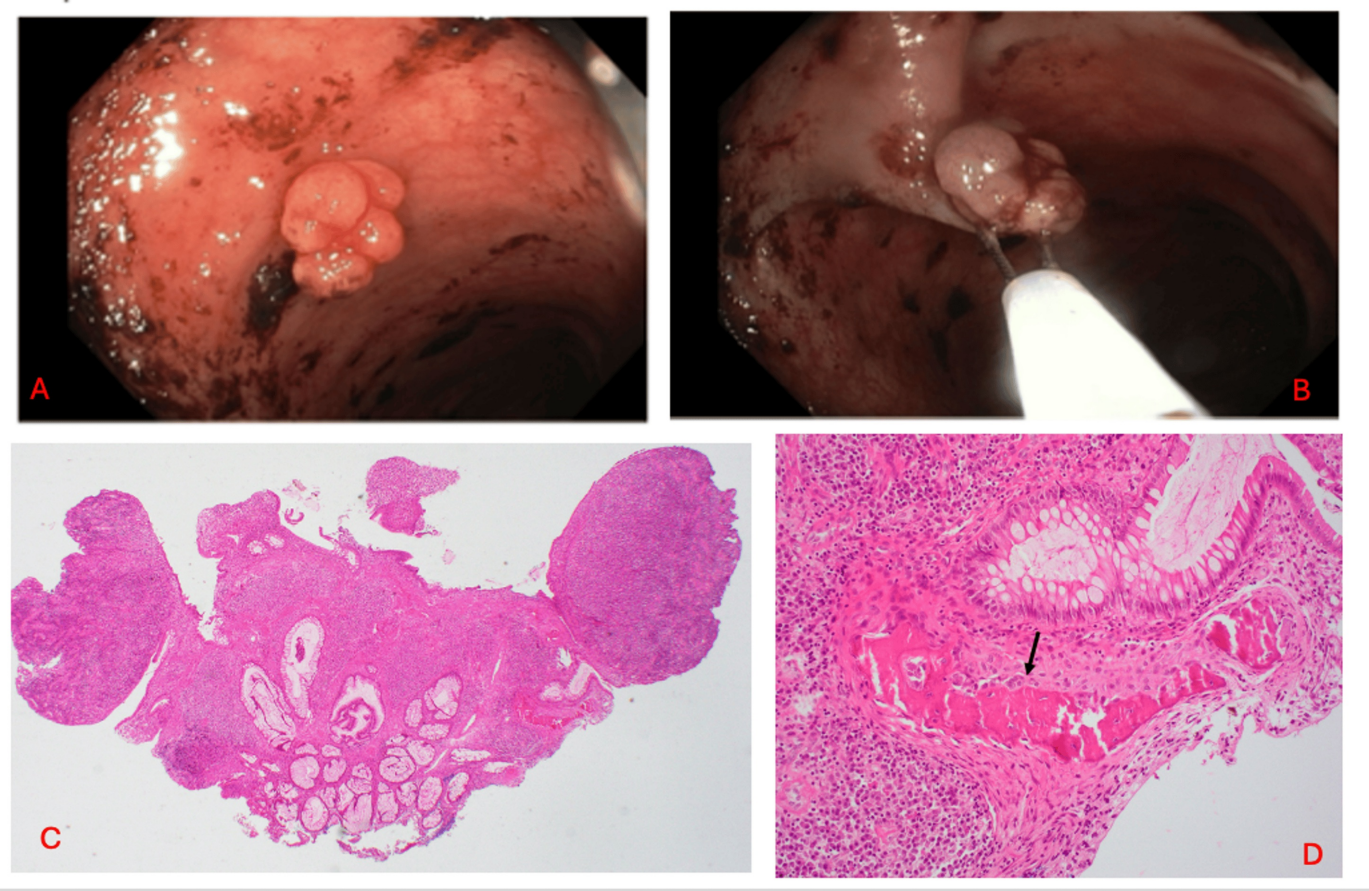
(A) Colonic polyp; (B) Snare polypectomy; (C) Juvenile polyp; (D) Osseous metaplasia

Upper gastrointestinal endoscopy and colonoscopy were subsequently performed. The colonoscopy was normal except for a small pedunculated polyp measuring less than 5 mm in the proximal transverse colon (Figures 1A, 1B). The base of the polyp was injected with 0.2 mL of epinephrine (1:100,000) in two quadrants to elevate the lesion. Hot snare polypectomy was then performed without complications or bleeding.

Histopathological examination demonstrated features consistent with a juvenile polyp, including dilated cystic glands and osseous metaplasia within the lamina propria (Figures 1C, 1D).

## Discussion

Metaplasia refers to the replacement of one differentiated mature cell type by another differentiated mature cell type, typically in response to chronic inflammation [1]. In our case, heterotopic ossified bone formation was identified within gastrointestinal tissue on histopathological examination.

Although osseous metaplasia is relatively common in sites such as the nasopharynx and endocervix, it is rarely observed in the gastrointestinal tract, with a reported prevalence of less than 1% [5]. A limited number of cases have been described in both benign and malignant lesions, including rectal adenocarcinoma and juvenile polyps [4,5].

In 2012, Veits et al. reported a 77-year-old patient presenting with rectal bleeding whose colonoscopy demonstrated proctosigmoiditis, while histology revealed active inflammation with metaplastic bone formation [2]. A literature review of pediatric gastrointestinal cases with osseous metaplasia identified 11 reported cases involving rectal polyps in children aged four to 17 years, most of whom presented with rectal bleeding [3].

Our case differs in that the lesion was located in the transverse colon rather than the rectum, and the patient presented primarily with abdominal pain rather than rectal bleeding.

The underlying pathogenesis of osseous metaplasia remains uncertain. Several mechanisms have been proposed, including local osteogenic stimulation leading to osteoblast differentiation and extracellular matrix production [6]. Another hypothesis suggests that mesenchymal cells undergo transformation into osteoblasts under the influence of epithelial-derived mediators, including bone morphogenetic proteins (BMP)-5 and BMP-6 produced by epithelial cells, as well as BMP-2 and BMP-4 secreted by surrounding fibroblasts [7].

## Conclusions

This case describes a rare occurrence of osseous metaplasia in a juvenile polyp located in the transverse colon, whereas previously reported pediatric cases predominantly involved rectal lesions. Although osseous metaplasia is generally considered a benign histopathological finding, its exact pathogenesis and long-term clinical significance remain unclear. Awareness of this rare phenomenon is important for gastroenterologists and pathologists to avoid misinterpretation and to contribute to the growing literature regarding its possible etiological mechanisms and prognostic implications. Additional pediatric case reports and long-term follow-up studies are needed to better understand whether this finding carries any clinical relevance or risk of recurrence.
